# Automated detection of cognitive impairment in clinical practice

**DOI:** 10.1007/s00415-024-12444-8

**Published:** 2024-06-04

**Authors:** Robyn M. Busch, Olivia Hogue, Abagail F. Postle, Darlene P. Floden

**Affiliations:** 1 Department of Neurology and Epilepsy Center, Neurological Institute, Cleveland Clinic, Cleveland, OH USA; 2 Center for Neurological Restoration, Neurological Institute, Cleveland Clinic, Cleveland, OH USA; 3 Department of Quantitative Health Sciences, Lerner Research Institute, Cleveland Clinic, Cleveland, OH USA

**Keywords:** Technology, Computerized, Automated, Memory, Cognition, Screening, Brief Assessment of Cognitive Health, BACH

## Abstract

**Objective:**

Cognitive impairment is now recognized as an impending public health crisis. About one-third of adults are concerned about their cognition, and the prevalence of objective cognitive impairment is much higher among those with neurological disorders. Existing screening tools are narrowly focused on detecting dementia in older adults and must be clinician-administered and scored, making them impractical for many neurology practices. This study examined the utility of a brief, self-administered, computerized cognitive screening tool, the Brief Assessment of Cognitive Health (BACH), in identifying cognitive impairment in adults.

**Methods:**

912 adults (ages 18–84) completed BACH and a neuropsychological battery. Multivariable models were developed to provide a BACH index score reflecting the probability of cognitive impairment for individual patients. Predictive accuracy was compared to that of the Montreal Cognitive Assessment (MoCA) in a subset of 160 older adults from a Memory Disorders clinic.

**Results:**

The final multivariable model showed good accuracy in identifying cognitively impaired individuals (*c* = 0·77). Compared to MoCA, BACH had superior predictive accuracy in identifying older patients with cognitive impairment (*c* = 0·79 vs. 0·67) as well as differentiating those with MCI or dementia from those without cognitive impairment (*c* = 0·86 vs. *c* = 0·67).

**Conclusions:**

Results suggest that cognitive impairment can be identified in adults using a brief, self-administered, automated cognitive screening tool, and BACH provides several advantages over existing screeners: self-administered; automatic scoring; immediate results in health record; easily interpretable score; utility in wide range of patients; and flags for treatable factors that may contribute to cognitive complaints (i.e., depression, sleep problems, and stress).

**Supplementary Information:**

The online version contains supplementary material available at 10.1007/s00415-024-12444-8.

## Introduction

Cognitive impairment is now recognized as an impending public health crisis [1]. Approximately one in three adults in the general population endorse subjective cognitive complaints [2–4], and the prevalence of cognitive impairment increases steadily over the lifespan [5, 6]. Disorders like Alzheimer’s disease often take center stage in discussions of population prevalence of cognitive impairment, but cognitive deficits occur as part of many developmental and acquired conditions in diverse neurological and non-neurological disorders and can significantly impact the patient’s functional status and treatment. While marked cognitive impairments may be detected in brief interactions, identifying milder forms of impairment or decline is a major challenge for physicians given shrinking time and resources available for direct patient care.

There are several available cognitive screening tools (e.g., Mini-Mental State Examination, Montreal Cognitive Assessment, and Mini-Cog), which were developed to detect dementia in older adults and thus have limited sensitivity in identifying milder forms of cognitive impairment. Further, these measures must be clinician-administered and scored, limiting feasibility of use in busy clinical practices. The overwhelming need for a more practical method of cognitive screening is well recognized. Unfortunately, the vast majority of available instruments do not have acceptable psychometric properties (e.g., reliability, validity) and fail to meet published standards for clinical use [7].

The Brief Assessment of Cognitive Health (BACH) was developed to provide a rapid, reliable, low resource method for clinicians to identify cognitive impairment in adult patients. It is a fully automated, computerized cognitive screening tool that can be completed independently by patients in multiple clinical settings or at home. The test is automatically scored and integrated into the medical record with results immediately available and easily added to clinical documentation. The BACH was rigorously developed to comply with standards recommended by American Academy of Clinical Neuropsychology [7] and took a unique approach to cognitive screening. Most existing screening tools have relatively easy stimuli (e.g., remember 3 words, copy a simple figure), so that failure on only a few items is indicative of impairment. In contrast, the BACH cognitive subtests were designed to be difficult to prevent ceiling effects that plague existing screening measures and to increase sensitivity to mild cognitive impairments and small changes in cognition over time.

The BACH includes a brief history questionnaire and a depression screen, to identify non-neurological factors that may contribute to cognitive inefficiencies and subjective complaints, and two complex memory measures that have been validated against gold-standard neuropsychological measures with good test–retest reliability [8]. Finally, the BACH has built-in validity indicators to identify test results that may not reflect true abilities. The primary goal of this study was to examine the utility of the BACH in identifying cognitive impairment on comprehensive neuropsychological evaluation. Study aims were to develop multivariable models using information collected with the BACH screening tool to estimate the probability of cognitive impairment for individual patients, and compare predictive accuracy of the BACH in identifying mild cognitive impairment (MCI) or dementia to that of the Montreal Cognitive Assessment (MoCA).

## Methods

### Standard protocol approvals and patient consents

This was an IRB-approved prospective cohort study. All participants were prospectively recruited between October 2017 and December 2019 and provided written informed consent for study participation.

### Participants

Participants were a diverse sample of adults, ages 18 and over. Approximately half of participants (*n* = 451) were Cleveland Clinic (Cleveland, Ohio) patients who were referred for neuropsychological testing as part of routine clinical care. Patients were referred from Memory Disorders (*N* = 160, 35%), General Neurology (*N* = 87, 19%), Movement Disorders (*N* = 53, 12%), Epilepsy (*N* = 43, 10%), Multiple Sclerosis (*N* = 23, 5%), Brain Tumor (*N* = 17, 4%), and Other = 68 (15%). The other half (*n* = 461) were adult patients not referred for cognitive testing, adult friends or family members of patients, or hospital employees. All participants completed the BACH and a full clinical neuropsychological evaluation (patients recruited from Neuropsychology Clinic) or a brief cognitive evaluation for research purposes (other patients, friends, family, and employees). A parking validation, gift card, or research stipend was provided to participants to compensate them for their time completing the study measures.

### Measures

#### Brief assessment of cognitive health (BACH)

**Fig. 1:**
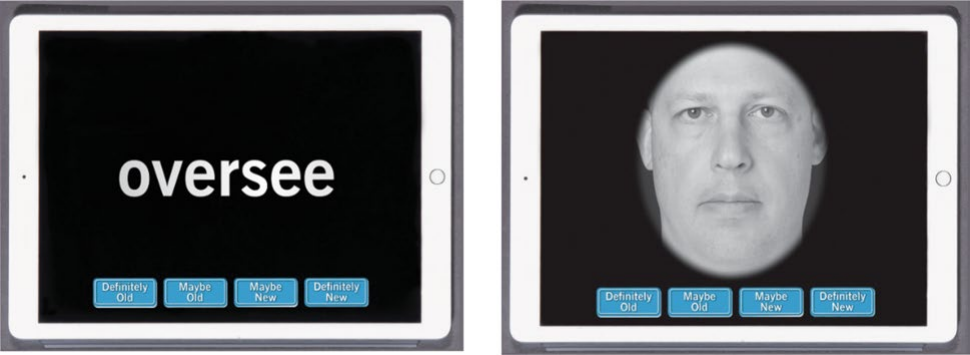
Sample stimuli from the Words and Faces Memory subtests

The BACH is a self-administered, computerized cognitive screening measure that patients can complete in a waiting room, office, or at home (all participants in this study completed the BACH in the hospital setting). Auditory test instructions are provided via speaker or over-the-ear headphones, and participants are instructed to adjust the audio to a comfortable level before they begin the assessment. The test consists of a brief history questionnaire (e.g., demographics, medical history), a validated depression screening measure (8-item Patient Health Questionnaire; PHQ-8), and two Memory subtests [8], all completed on an iPad. The questionnaire includes items pertaining to sleep duration and quality (based on the National Sleep Foundation’s sleep time duration recommendations for adults [9]) and stress level. The Memory subtests include verbal (Words) and nonverbal (Faces) ‘remember-know’ recognition memory tests where participants study 20 words and 20 faces and then are shown those stimuli interspersed with 20 new stimuli and asked whether they remember the stimulus and how confident they are in that memory. Reaction times and accuracy are recorded for each item. Detailed information regarding the development and initial validation of the Memory subtests is reported elsewhere [8], and exemplars of the memory stimuli are provided in Fig. 1.

#### Neuropsychological battery

Patients recruited through the Neuropsychology Clinic completed comprehensive neuropsychological evaluations that included measures assessing the following cognitive domains: attention/working memory, processing speed, language, visuospatial functioning, executive functioning, and memory. Family members, friends, employees, and other Cleveland Clinic patients were recruited via study flyers and completed a brief research battery that included one to two measures in each of the same cognitive domains. All participants had adequate vision and hearing to complete the administered battery. Whenever possible given clinical demands, the BACH and clinical neuropsychological measures were administered in a counterbalanced order, while BACH and the research battery were always counterbalanced. A subset of older patients (60 + years) who were referred for evaluation through the Memory Disorders clinic also completed the MoCA as part of their clinical evaluations. Supplemental Table 1 summarizes the neuropsychological measures administered for research or clinical purposes.

**Table 1:** Sample demographics

		Full sample *n* = 912	Clinical NP battery *n* = 451	Research battery *n* = 461
Age, mean (SD)		53·0 (15·8) Range 18–84	56·7 (15·4) Range 18–84	49·5 (15·3) Range 18–84
Age group, *n* (%)	18–29	95 (10.4)	39 (8.7)	56 (12.2)
	30–39	117 (12.8)	31 (6.9)	86 (18.7)
	40–49	133 (14.6)	47 (10.4)	86 (18.7)
	50–59	202 (22.2)	106 (23.5)	96 (20.8)
	60–69	225 (24.7)	129 (28.6)	96 (20.8)
	70–79	123 (13.5)	89 (19.7)	34 (7.4)
	80–84	17 (1.9)	10 (2.2)	7 (1.5)
Female, *n* (%)		548 (60·1)	257 (57·0)	291 (63·1)
Race, *n* (%)	White	675 (74·0)	412 (91·4)	263 (57·1)
	Black	211 (23·1)	32 (7·1)	179 (38·8)
	Other, including multiracial and not reported	26 (2·9)	7 (1·55)	19 (4·1)
Hispanic/Latino, *n* (%)		24 (2·6)	9 (2·0)	15 (3·3)
Education, mean (SD)		14·3 (2·7) Range 3–20	14·5 (2·7) Range 7–20	14·1 (2·7) Range 3–20
Employment, *n* (%)	Full-time	320 (35·1)	126 (27·9)	194 (42·1)
	Part-time	90 (9·9)	46 (10·2)	44 (9·5)
	Retired	249 (27·3)	165 (36·6)	84 (18·2)
	Student	19 (2·1)	11 (2·4)	8 (1·7)
	Disabled	125 (13·7)	62 (13·8)	63 (13·7)
	Unemployed	109 (12·0)	41 (9·1)	68 (14·8)

*NP* neuropsychological

### Impairment classification

To ensure objectivity and consistency across clinical and research batteries, impairment on each cognitive test in the neuropsychological battery was defined as performance < 1·5 standard deviations below the normative mean. Patients impaired on at least one test in at least two cognitive domains or at least two tests in a single cognitive domain were classified as “cognitively impaired” for purposes of this study. All others were classified as “cognitively intact”. For domain-specific analyses, patients had to have reduced performance ( < 1·5 SD below the mean) on at least two tests within the domains of executive functioning or memory or on at least one test within the domains of attention, processing speed, language, or visuospatial skills to be classified as “impaired” for that domain. The decision to base the latter classification on one or more subtests was due to the limited number of measures in the research battery used to assess these latter domains. The MoCA test was not used to determine impairment.

### Statistical analyses

Descriptive statistics summarizing demographic and medical characteristics were calculated for the sample and are presented as means with standard deviation for normally-distributed continuous variables, medians with interquartile range for skewed continuous variables, and frequencies with proportion for categorical variables. Normality was assessed visually using Q–Q plots. Any impairment and domain-specific impairment were considered as separate binary outcomes.

#### Model building

Multivariable logistic regression models were developed to identify any cognitive impairment using an a priori-defined list of candidate variables from the BACH screening tool, including BACH Memory subtest scores (Faces and Words) and demographic, medical, sleep, stress, and mood items assessed via BACH questionnaires. Select medical history questionnaire endorsements were expected to increase probability of an objective cognitive problem (i.e., cognitive impairment on neuropsychological measures) and were grouped for model-building (e.g., previous stroke, traumatic brain injury, and seizures). Similarly, select medical history questionnaire endorsements were expected to increase subjective cognitive complaints (i.e., patient self-report of a cognitive problem) but not increase the probability of objective impairment (i.e., cognitive impairment on neuropsychological measures) (e.g., mood and psychiatric diagnoses, sleep disorders, etc.).

To select the best-fitting, most parsimonious model, Harrell’s stepdown procedure was used [10]. This method prioritizes the ability of the model to discriminate between impaired and non-impaired patients and removes predictor variables sequentially according to their impact on discrimination. Continuous variables were assessed for linearity by plotting against the logit, with the intention to perform variable transformation or fit models using restricted cubic splines if necessary, but this was ultimately not needed.

#### Model evaluation

The discriminatory ability of each model was evaluated using a bootstrap-corrected concordance (c) statistic [10]. The c-statistic, equivalent to the area under the receiver-operating characteristic curve (ROC), ranges from 0·5 to 1·0, with a value of 0·5 indicating that the model performs no better than chance, and a value of 1·0 indicating that the model correctly classifies 100% of patients. Values of 0·70–0·79 are considered good, and values of 0·80 to 0·89 are considered excellent. Model calibration was evaluated by plotting assigned probabilities against observed impairment and deriving a confidence band, known as the Giviti calibration belt [11].

Multivariable logistic regression models use patient-specific information to generate a probability value for each patient to indicate likelihood the patient would be considered impaired after undergoing formal neuropsychological testing. The relationship between probability of cognitive impairment, as determined by the multivariable models, and degree of cognitive impairment on neuropsychological evaluation was examined. The distribution of model-assigned probabilities was stratified by the number of impaired neuropsychological tests as well as by the number of impaired cognitive domains.

#### Predictive accuracy for MCI/dementia clinical diagnosis

Data from a subset of patients seen in the Memory Disorders clinic were used to compare the predictive accuracy of the BACH (c-statistic from continuous probability score) and MoCA (c-statistic using the recommended cutoff of 26 to indicate impairment) in identifying presence/absence of MCI or dementia based on Memory Disorders Consensus Conference diagnosis.

### Software

Study data were managed using REDCap electronic data capture tools [12, 13]. Analyses were conducted using SAS Studio v. 3.3 and R Studio package “rms.”[14] Models were not generated to test hypotheses and therefore no *p*-values are provided.

## Results

### Sample characteristics

The sample consisted of 912 adults (60% female, 74% White), all of whom were able to complete the measure independently without assistance in a median of 16 min (IQR 14–19, range 11–36 min). Participants were 53 years old on average (range 18–84 years) and had approximately 14·3 years (SD = 2·7) of education. A summary of the demographic characteristics of the sample overall and separately by type of cognitive battery completed is provided in Table 1. More than half the sample reported a previous diagnosis of at least one condition associated with subjective rather than objective cognitive complaints, while about a quarter reported a previous diagnosis of at least one condition that commonly presents with objective cognitive impairments. A more detailed description of self-reported medical conditions can be found in Table 2.

**Table 2:** Self-reported medical conditions

	*n* (%)
Any condition associated with objective cognitive impairment^*^	259 (28·4)
Any condition associated with subjective cognitive impairment^†^	524 (57·5)
Healthy^§^	197 (21·6)
ADHD	65 (7·1)
Learning disability	91 (10·0)
Hypertension	222 (24·3)
Diabetes	124 (13·6)
COPD/Emphysema	35 (3·8)
Heart Disease	75 (8·2)
Sleep Disorder	220 (24·1)
Migraines	185 (20·3)
Seizures/Epilepsy	114 (12·5)
Stroke/TIA/aneurysm	54 (5·9)
Movement disorder	50 (5·5)
Brain tumor	25 (2·7)
Other cancer	171 (18·8)
Cerebral palsy	2 (0·2)
Traumatic brain injury	30 (3·3)
Meningitis/encephalitis	9 (1·0)
Multiple sclerosis	20 (2·2)
Normal pressure hydrocephalus	3 (0·3)
HIV/AIDS	5 (0·6)
Mild cognitive impairment	11 (1·2)
Dementia	4 (0·4)
Depression/Anxiety	355 (38·9)
Other mental health condition	47 (5·2)
Chronic fatigue	39 (4·3)
Fibromyalgia	46 (5·0)

^*^Includes endorsement of any of the following: brain tumor, stroke/TIA/aneurysm, traumatic brain injury, movement disorder, seizures/epilepsy, multiple sclerosis, normal pressure hydrocephalus, meningitis encephalitis

^†^ Includes endorsement of any of the following: depression/anxiety, sleep disorder, chronic fatigue, fibromyalgia, migraines, other mental condition

^§^ Precludes endorsement of any of the conditions associated with objective or subjective cognitive impairment in addition to the following: other cancer, COPD/emphysema, heart disease, HIV/AIDS. Hypertension and diabetes endorsements allowed

The subsample of older adults used to compare the predictive accuracy of the BACH and the MoCA consisted of 160 patients who underwent multidisciplinary assessment in the Memory Disorders clinic (50% female, 88% white). Participants were 65 years old on average (range 60–84 years) and had approximately 15·1 years (SD = 2.9) of education. In this sample, 11 (7%) had a clinical consensus diagnosis of dementia, 46 (29%) had a clinical consensus diagnosis of MCI, and 103 (64%) did not have evidence suggesting the presence of a neurodegenerative condition. Consensus diagnosis was made based on results of multidisciplinary evaluations, which include neurological examination, neuropsychological assessment, and neuroimaging as well as results of any other testing that was done to clarify diagnosis (e.g., labs, CSF testing, amyloid imaging, PET, etc.).

### Cognitive impairment on neuropsychological assessment

A total of 310 participants (34%) demonstrated some type of cognitive impairment on the neuropsychological assessment. Memory impairment was most common (35·5% impaired on at least one memory test) followed by executive function (22·6%) and language impairment (21·6%). Processing speed (12%) and attention impairments (8%) were less commonly observed.

### Multivariable model to identify cognitive impairment

**Fig. 2:**
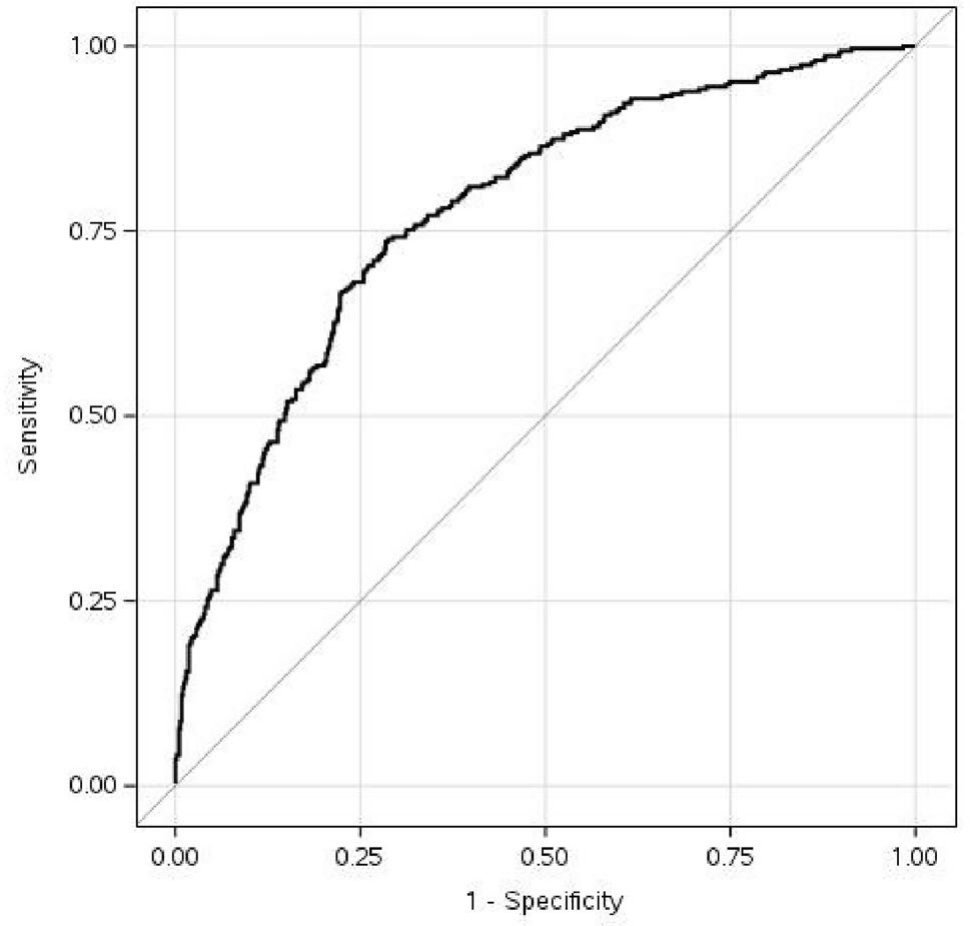
ROC curve

**Fig. 3:**
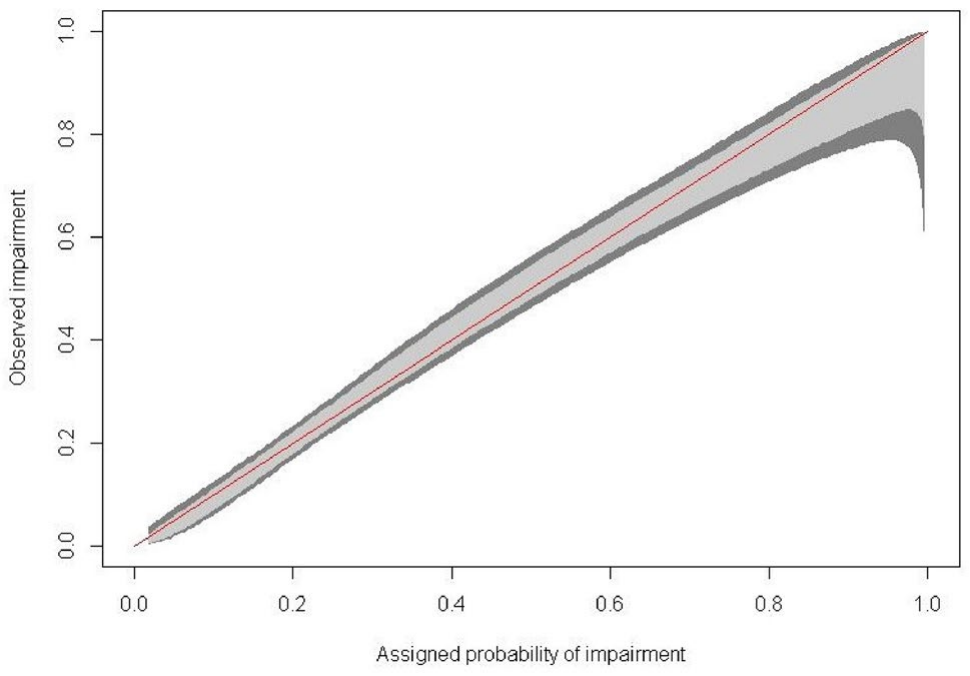
Model calibration. A perfectly calibrated model has predicted probabilities that match the observed impairment in the sample, creating a 45 degree line with narrow and uniform confidence bands. This indicates assigned probabilities are not subject to systematic under- or over-estimates and the model does not suffer from over-fitting. For both models, the 80% (light gray) and 90% (dark gray) confidence bands neither approach nor cross the ideal 45 degree line, indicating that model predictions are reliable estimates of actual probability of impairment. Confidence bands are relatively narrow and even throughout, indicating that uncertainty associated with predictions is consistent and minor

The final multivariable model included eight variables: Memory subtest performance, depression score on the PHQ-8, median correct and incorrect reaction times, self-reported condition associated with subjective cognitive impairment (e.g., anxiety, sleep disorder), self-reported condition associated with objective cognitive impairment (e.g., seizure disorder, multiple sclerosis), education, and the interaction between Memory score and depression status. The model achieved an optimism-corrected c-statistic of 0·77. This indicates that when presented with two individuals, one with a cognitive impairment and one without, the model would correctly assign the impaired patient a higher probability of impairment 77% of the time. The ROC curve for the model is depicted in Fig. 2. Model calibration was excellent, as displayed by the Giviti calibration belt shown in Fig. 3. The median model-assigned probability of impairment among those NOT classified as impaired was 23% (IQR 13–35%). The median model-assigned probability of impairment among those classified as impaired was 45% (IQR 30–66%). Model performance in identifying domain-specific cognitive impairment is provided in Table 3.

**Table 3:** BACH discrimination (c-statistic) by cognitive domain

	*c*-statistic
Any Cognitive Impairment	0·77
Attention Impairment	0·67
Processing Speed Impairment	0·66
Language Impairment	0·62
Visuospatial Impairment	0·70
Executive impairment	0·72
Memory impairment	0·75

Impairment classification in the Executive and Memory domains was based on impairment on 2 or more impaired tests within the given domain. Impairment classification for Attention, Processing Speed, Language, and Visuospatial was based on impaired performance on 1 or more tests within the given domain

### Model probabilities and degree of objective cognitive impairment

**Fig. 4:**
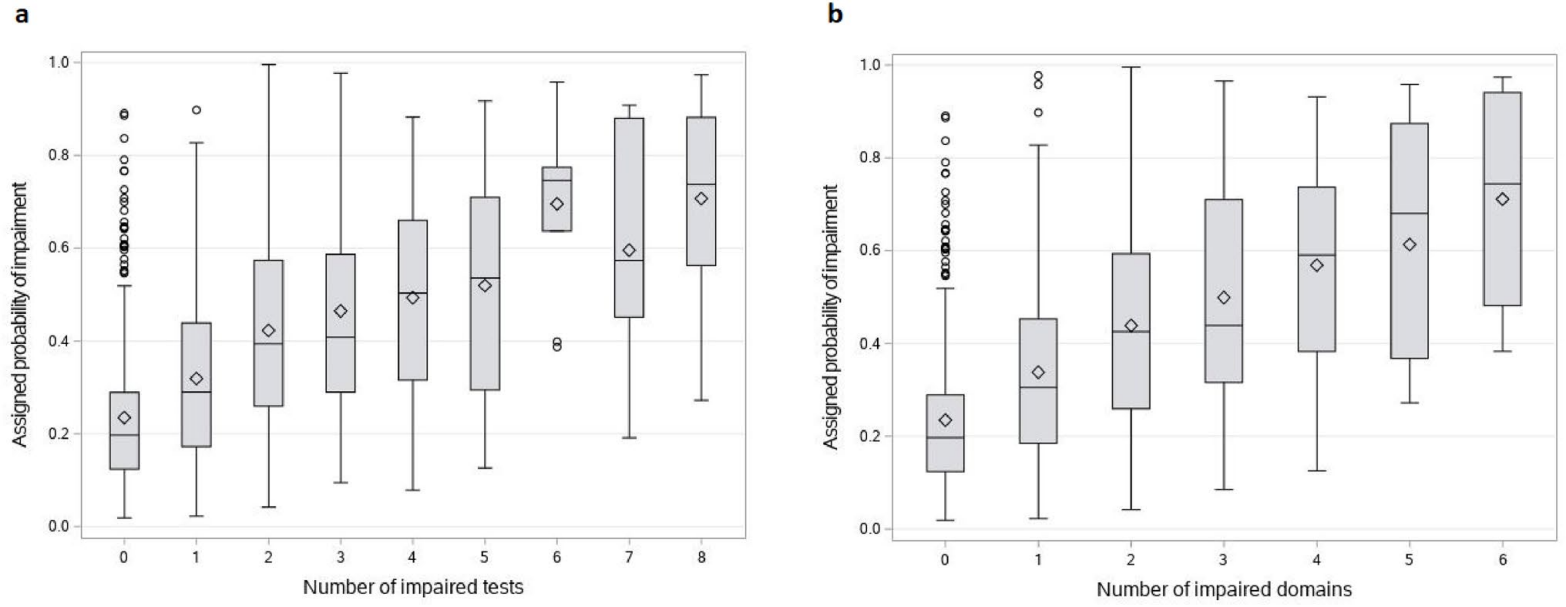
Model probabilities and degree of impairment on neuropsychological measures. **a** Distribution of assigned probabilities stratified by number of impaired neuropsychological measures. Due to the highly skewed distribution, patients impaired on eight or more tests are all included in the box labeled 8. **b** Distribution of assigned probabilities stratified by number of impaired cognitive domains. Boxes contain the middle 50% of scores, with each horizontal line representing the median. Each diamond is the mean. Whiskers extend to q1 and q3, with each dot representing an outlier. The figures show that as the number of impaired tests or cognitive domains increases, the assigned probability of impairment also increases

Figure 4a shows the distribution of assigned probabilities using the BACH model stratified by number of impaired neuropsychological tests. The figure shows that as the number of impaired tests increases, the assigned probability of impairment also increases. A similar positive relationship was observed for the number of impaired cognitive domains (Fig. 4b).

### Predictive accuracy for MCI/dementia clinical diagnosis

In the subset of 160 Memory Clinic patients, BACH had superior predictive accuracy compared to the MoCA in identifying cognitive impairment based on neuropsychological testing alone (*c* = 0·79 vs. 0·67) as well as based on the clinical consensus diagnosis of MCI or dementia versus no cognitive impairment (*c* = 0·86 vs. *c* = 0·67).

## Discussion

This study demonstrates the utility of the Brief Assessment of Cognitive Health as an automated, self-administered tool to screen for cognitive impairment in adults with a wide range of ages and abilities. A multivariable model, including the BACH Memory subtests and questionnaire responses, assigns a higher probability of impairment to the cognitively impaired individual 77% of the time.

As noted, the BACH Memory subtests are rather unique compared to memory components of other cognitive screening tools in that they were designed to be difficult to prevent ceiling effects, permitting greater sensitivity to impairment in adults with higher ability levels[8]. Most existing screening measures were developed and validated primarily for use in older individuals and consist of a few relatively easy items, so that when a patient does poorly, cognitive impairment is almost certainly present. A significant limitation of such measures is difficulty identifying individuals with mild cognitive deficits, particularly younger adults or those with high premorbid levels of functioning. The BACH was designed with the goals of identifying any cognitive impairment, even if it is relatively mild, and to identify cognitive impairment in adults of any age with any medical history (i.e., disease agnostic), so that it could be used in diverse clinical practices.

While the Memory subtests show strong correlations with performance on traditional neuropsychological measures of memory[8], careful consideration was given during test development to select test stimuli and response format/measurements that capture impairments in other cognitive domains as well [8]. Many cognitive screening tests overemphasize memory dysfunction while neglecting other cognitive domains that may be affected early in disease processes. Thus, our goal was to develop a tool that would capture impairment in a range of cognitive domains. Consistent with this goal, our results show that the BACH does a good job of identifying patients with cognitive impairments in nearly any cognitive domain. That said, it is important to note that BACH was designed as a general cognitive screening measure to predict impairment in *any* cognitive domain, and only the algorithm for *any* cognitive impairment (*c* = 0.77) is provided in the BACH output that goes to clinicians. While we have provided the c-statistics to demonstrate BACH’s ability to predict impairment in specific cognitive domains (i.e., attention, processing speed, language, visuospatial skills, executive function, and memory), this screening tool should not be used to infer domain-specific impairments. Not surprisingly, given the structure of this screening tool and linguistic demands for participation, prediction of language impairment yielded the lowest concordance statistic. As such, this screening tool is not recommended when a selective language deficit is suspected.

The BACH is also different than many screening measures in that it concurrently screens for non-neurological factors that may impact cognitive functioning or contribute to subjective cognitive complaints. Depressive symptoms and sleep disturbance are often associated with inefficiencies in cognition and subjective cognitive complaints, particularly in the domains of attention, processing speed, executive function, and learning/memory, which may normalize following symptom remission [15–19]. Stress, particularly prolonged stress, can also have a marked impact on cognitive functioning [20]. Thus, we believe that it is important to assess these factors when screening cognition to aid clinical decision-making. Patients with significant depressive symptoms or sleep difficulties may benefit from consultation with a mental health provider or sleep specialist to address and treat these issues prior to neuropsychological assessment or cognitive diagnosis.

Not surprisingly given the cognitive demands of the Memory subtests, Memory subtest performance was the most important variable in predicting cognitive impairment on neuropsychological testing with lower BACH Memory scores yielding higher probability of cognitive impairment. Reaction times on the Memory items also contributed to our calculation of probability of impairment. Specifically, longer reaction times to correct responses contributed an incremental increase to the probability of cognitive impairment while longer reaction times to incorrect responses contributed to a decrease in probability of cognitive impairment. This is consistent with the cognitive neuroscience literature showing that that reaction times reflect an interaction between accuracy and strength of a memory [21].

There were several other variables that served to improve predictive accuracy in our multivariable model. Consistent with our expectations, medical conditions associated with objective cognitive impairment (e.g., stroke, traumatic brain injury, and seizures) contributed an increase in the probability of cognitive impairment, while medical conditions associated with subjective cognitive impairments (e.g., mood or psychiatric diagnosis, and sleep disorder) yielded a decrease in the probability of cognitive impairment. Higher education (i.e., ≥ 16 years) also decreased the probability of cognitive impairment. There is an extensive literature demonstrating that higher educational attainment is associated with higher cognitive test scores and better cognitive outcomes following brain injury or insult [22–24]. Finally, depression score on the PHQ-8, reflecting current depressive symptomatology, modified the impact of BACH score on assigned probability; the impact of BACH score on impairment probability is stronger among respondents with low PHQ-8 scores, when compared to those with elevated depressive symptoms per PHQ-8.

The final multivariable prediction model yields a probability of cognitive impairment on a continuous scale, rather than a simple dichotomous outcome (i.e., impaired vs. not impaired). This permits the end-user to interpret results in the unique clinical context of a particular patient. If the BACH output also suggests that the patient is depressed, reporting significant stress, or may have a sleep disorder, the clinician may incorporate this into their treatment decisions and refer for additional evaluations before pursuing a formal neuropsychological evaluation. Ultimately, the BACH was designed to be a decision-making tool for clinicians rather than a diagnostic measure. Thus, it can be incorporated into clinical practice in whatever way is most useful given the specific practice and patient population. For example, physicians treating patients with progressive disorders that involve cognition may wish to use this tool to screen cognition in all newly diagnosed patients and to track changes over time, while physicians seeing patient groups that often present with vague, subjective complaints (i.e., concussion, POTS, fibromyalgia) may wish to use this tool to determine whether more formal cognitive assessment is warranted.

**Fig. 5:**
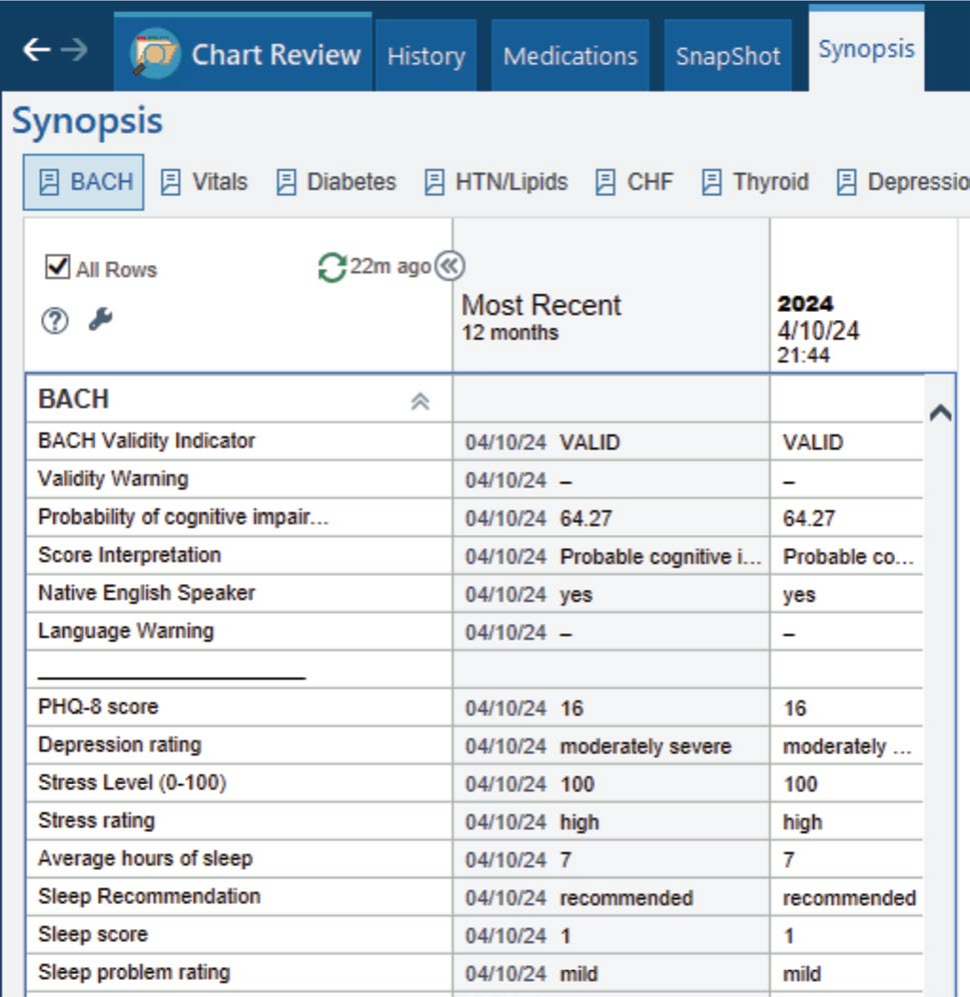
Representative display of test results provided to the ordering physician via the electronic health record

To facilitate clinical use, BACH has been integrated into the Epic electronic medical record system. The BACH can be ordered directly from the patient’s chart similar to other EHR workflows. The patient can complete the screening measure either prior to, during, or following their clinical visit, and results are provided to the clinician instantaneously within the patient’s medical record (Fig. 5). A web-based version of the BACH has recently been developed and is currently being validated for remote cognitive screening. A smart phrase has also been created to generate a written summary of the BACH results that can be incorporated into the visit documentation or progress note.

There are several limitations of the study that we hope to address in future research. These include data generated from a single center in only English-speaking adults, limited ethnoracial diversity of participants, and integration of BACH results into only one electronic health record system (i.e., Epic). Nevertheless, our findings demonstrate that self-administered computerized cognitive screening is feasible for use in adults to identify those who may require more formal assessment.

## Conclusions

In summary, this study provides data to support the clinical utility of the BACH as a screening measure for cognitive impairment in adults of all ages and a wide range of abilities, regardless of medical history/condition. This self-administered, automated, computerized tool can be completed in < 15 min, automatically generates a probability of cognitive impairment, and screens for non-neurologic factors that may be contributing to cognitive complaints/difficulties. BACH identifies individuals with cognitive impairment with 77% accuracy and outperforms a leading paper-and-pencil screen in detecting clinically diagnosed MCI and dementia. The measure has been integrated with the Epic electronic medical record to provide instantaneous results to the clinician and to facilitate documentation requirements. Implementation studies are underway to determine the best way to integrate BACH cognitive screening into clinical workflows. Future research will seek to examine the utility of the BACH in specific patient populations and to translate the BACH for use in patients who primary language is not English.

### Supplementary Information

Below is the link to the electronic supplementary material.

Supplementary file1 (DOCX 14 KB)

## Data Availability

The datasets analyzed in the current study are not publicly available because of restricted access, and model coefficients are not provided due to a pending patent application. However, further information about the datasets is available from the corresponding author on reasonable request.
