# Supplementary material for: Automated detection of cognitive impairment in clinical practice
Source: J Neurol. 2024 Jun 4;271(8):5187–96. doi: 10.1007/s00415-024-12444-8 (PMC11319487; doi:10.1007/s00415-024-12444-8)
Supplement: Supplementary file 1 — Supplementary file1 (DOCX 14 KB) [file 415_2024_12444_MOESM1_ESM.docx]

**Supplemental Table 1. Neuropsychological Test Measures**

| **Domain** | **Measures** | **Completed for Research Battery (n=461)** | **Completed for Clinical Purposes (n=451)** |
| --- | --- | --- | --- |
| Language | Boston Naming Test | -- | 451 |
|  | Boston Naming Test – Short Form | 461 |  |
|  | Semantic Fluency - Animals | -- | 299 |
| Attention | Wechsler Scales – Digit Span | 461 | 317 |
| Processing Speed | Trail Making Test – Part A | 461 | 192 |
| Executive Function | Trail Making Test – Part B | 461 | 187 |
|  | Phonemic Fluency | -- | 410 |
|  | Wisconsin Card Sorting Test – Perseverative Errors | -- | 257 |
| Visuospatial | Wechsler Scales – Spatial Span | -- | 66 |
|  | Judgment of Line Orientation | -- | 382 |
|  | Judgment of Line Orientation – Short Form | 461 |  |
|  | Wechsler Scales – Block Design | -- | 213 |
|  | Wechsler Scales – Matrix Reasoning | -- | 103 |
| Verbal Memory | Rey Auditory Verbal Learning Test | 461 | 210 |
|  | Hopkins Verbal Learning Test | -- | 130 |
|  | California Verbal Learning Test | -- | 98 |
|  | Wechsler Scales III– Logical Memory | -- | 135 |
|  | Wechsler Scales III– Verbal Paired Associates | -- | 27 |
|  | Wechsler Scales IV– Logical Memory | -- | 290 |
| Visual Memory | Brief Visual Memory Test | -- | 249 |
|  | Wechsler Scales - Faces | 461 | 32 |
